# Durability and Mechanical Properties of Nano-SiO_2_ and Polyvinyl Alcohol Fiber-Reinforced Cementitious Composites Subjected to Saline Freeze–Thaw Cycles

**DOI:** 10.3390/ma17112542

**Published:** 2024-05-24

**Authors:** Lijun Wan, Yongqi Zhao, Maopei Yu, Ye Tian, Yipeng Wang

**Affiliations:** 1 School of Civil Engineering and Transportation, Northeast Forestry University, Harbin 150040, China; zhaoyongqi426@163.com (Y.Z.); yumaopei147@163.com (M.Y.); 2 Institute of Cold Regions Science and Engineering, Northeast Forestry University, Harbin 150040, China; 3 Heilongjiang Province Academy of Cold Area Building Research, Harbin 150080, China; tianli810@163.com (Y.T.); s923290570@163.com (Y.W.)

**Keywords:** nano-silica, polyvinyl alcohol fiber, durability properties, synergistic effect, fracture toughness

## Abstract

To investigate the effects of nano-SiO_2_ (NS) and polyvinyl alcohol (PVA) fibers on the durability and mechanical properties of cementitious composites subjected to saline freeze–thaw cycling, a series of PVA fiber-reinforced cementitious composite (PFRCC) specimens were prepared using various fiber contents, and a series of NS and PVA fiber-reinforced cementitious composite (NPFRCC) specimens were prepared using various combinations of NS and fiber contents. Durability and fracture toughness tests were subsequently conducted on the specimens after different numbers of saline freeze–thaw cycles. The results indicate that the degradation of material properties can be divided into slow and accelerated damage stages before/after 50 freeze–thaw cycles. The durability and fracture toughness of the specimen series tended to increase, then decrease with increasing NS and PVA contents, suggesting optimum levels. When the PVA fiber content was 0.5%, PFRCC specimens had the best durability after saline freeze–thaw cycles; when the NS and PVA fiber contents were 1.0% and 0.5%, respectively, NPFRCC specimens had the best durability and fracture properties, and the initiation toughness, destabilization toughness, and fracture energy after 100 saline freeze–thaw cycles were 120.69%, 160.02%, and 451.31%, respectively. The results of this study may guide future exploration of the durability and mechanical properties of concrete subjected to freeze–thaw action.

## 1. Introduction

Concrete is a widely used construction material today due to its good mechanical properties, excellent stability, and low cost. However, concrete also has the disadvantages of high brittleness and poor corrosion resistance at the same time, which seriously affects its application and service life in engineering structures. In Northeast China, when the temperature drops below 0 °C in winter, water infiltrating into concrete crevices undergoes a water–ice phase transition to produce many small cracks inside the structure [1,2,3]. Deicing salts are often used in winter due to the need to quickly remove snow and ice from roads and bridges. However, common deicing salts, usually NaCl and Cl-, penetrate the cement matrix through cracks and accelerate material strength damage under the action of freeze–thaw cycles [4,5]. Therefore, the development of cementitious composites with high durability and excellent mechanical properties is necessary to address the challenges faced by concrete applications in regions with negative temperatures.

Researchers have attempted to improve the frost resistance of concrete by increasing the compactness of the cement composite matrix. With the development of science and technology, the application of nanomaterials in engineering has gradually become widespread. Nanomaterials are ultrafine materials with a particle size of 1~100 nm, and the more mature nanomaterials include nano-SiO_2_ [6], nano-CaCO_3_ [7], nano-TiO_2_ [8], and carbon nanotubes [9]. Nano-SiO_2_ has high volcanic ash activity, which can quickly react with Ca(OH)_2_ and release a large amount of heat during hydration, thus significantly promoting the cement hydration reaction, generating a large number of reticulated dense C-S-H gels by consuming more hydration intermediates at an early stage, and optimizing the internal pore structure by making the C-S-H gels extend in a columnar direction. Meanwhile, due to the nucleation effect, nano-SiO_2_ can provide more C_3_A hydration active sites to promote cement hydration and thus significantly improve the mechanical properties of concrete as well as the degree of densification of the microstructure. [10], which is nowadays used to replace part of the cementitious materials to improve their durability and mechanical properties [11,12,13]. Chekravarty et al. [14] added a 3% cement mass of NS to determine its compressive strength after immersion in 5% NaSO_4_ for 90 d. The results showed that the compressive strength of NS concrete increased by 3.35% compared to normal concrete after the specimens were immersed in a salt solution for 90 days. Zhao et al. [15] added NS to recycled coarse aggregate concrete to determine its compressive strength, flexural strength, and other properties, and the results showed that the 2% of NS had the best modification effect on recycled aggregate concrete, and its compressive strength and flexural strength were increased by 31.8% and 33.2% compared with those before modification. Nazerigivi et al. [16] prepared four kinds of concrete specimens with different dosages of NS to determine their fracture toughness in different loading modes, and the results showed that the fracture toughness of concrete with a 0.5% NS dosage was the highest.

However, the enhancement of concrete durability by just adding NS is limited. In order to ensure that concrete still maintains better mechanical properties after freeze–thaw cycles, due to the bridging role of fibers in the cracks of the concrete matrix, it is of great significance to inhibit the formation and development of cracks under the action of freeze–thaw and further improve the durability and mechanical properties of concrete. In the process of continuous research, scholars have carried out a large number of studies on the addition of steel fibers [17], basalt fibers [18], and polypropylene fibers [8] to concrete, especially the development of concrete properties under different environmental conditions, which has become a popular research topic [19]. It is noteworthy that polyvinyl alcohol (PVA) fibers, which have high strength, modulus of elasticity, abrasion resistance, acid and alkali resistance, and do not react with hydration products, are receiving more and more attention nowadays [20]. The composite addition of NS and PVA fibers into concrete using the denser matrix achieved by NS can increase the encapsulation force on the fibers so that they can provide stronger bridging to enhance the durability and mechanical properties of concrete, and this is gradually becoming a popular research topic. Gao et al. [21] added NS with PVA fibers to mortar to test its shear resistance, and the results showed that specimens with a fiber content of 0.8% and an NS content of 2% had the best performance. Zhang et al. [22,23] tested the effect of NS and PVA fibers on the mechanical properties of mortar and found that specimens containing 0.6% PVA and 1.5% NS had a 43.9% higher compressive strength than unadulterated mortar specimens. Sun et al. [24] studied the effect of the addition of NS and PVA fibers on the hardening properties of recycled aggregate concrete and its mechanical properties. Fiber-recycled aggregate concrete hardening properties and microstructural changes reported the maximum compressive strength of recycled aggregate concrete containing 3% NS admixture and determined that the higher the NS content, the higher the split tensile strength of the specimens. Wang et al. [25] investigated the effect of NS with PVA fiber-reinforced cement composites on the durability of cement composites in complex environments, such as permeability and frost resistance. The results showed that the durability of the gel composites increased and then decreased with the increase in NS content, and the optimum NS content was 1.5%. Although a large number of studies have been carried out on NS and PVA fibers in mortar and concrete, these studies have mainly focused on compressive and shear strengths, as well as in water-freezing or sulfate environments, and there is still a lack of research on the freeze–thaw durability of NS and PVA fiber concrete in chloride salt environments. In addition, concrete fracture is a common phenomenon in the damage of cementitious composites, which can be used for structural design and safety evaluation. However, there are few studies on the fracture properties of NS with PVA fiber concrete in chloride salt environments.

In short, many scholars have studied the durability performance, basic mechanical properties, and reinforcement mechanisms of NS and PVA fibers on concrete from different perspectives. However, there are few studies on the durability as well as fracture properties of composite concrete under saline freeze–thaw cycle conditions. Therefore, in this study, cementitious composite specimens with a 0–2.5% mass fraction of NS and a 0.1–0.9% mass fraction of PVA fibers were subjected to 0, 25, 50, 75, and 100 freeze–thaw cycles in a 3.5% NaCl solution. The mass loss rate, dynamic modulus of elasticity, and compressive strength were determined to evaluate the effect of NS and PVA fibers on the durability of concrete. The effects of NS and PVA fibers on the fracture properties of concrete specimens after freeze–thaw cycles were also investigated.

## 2. Materials and Methods

### 2.1. Materials

**Figure 1:**
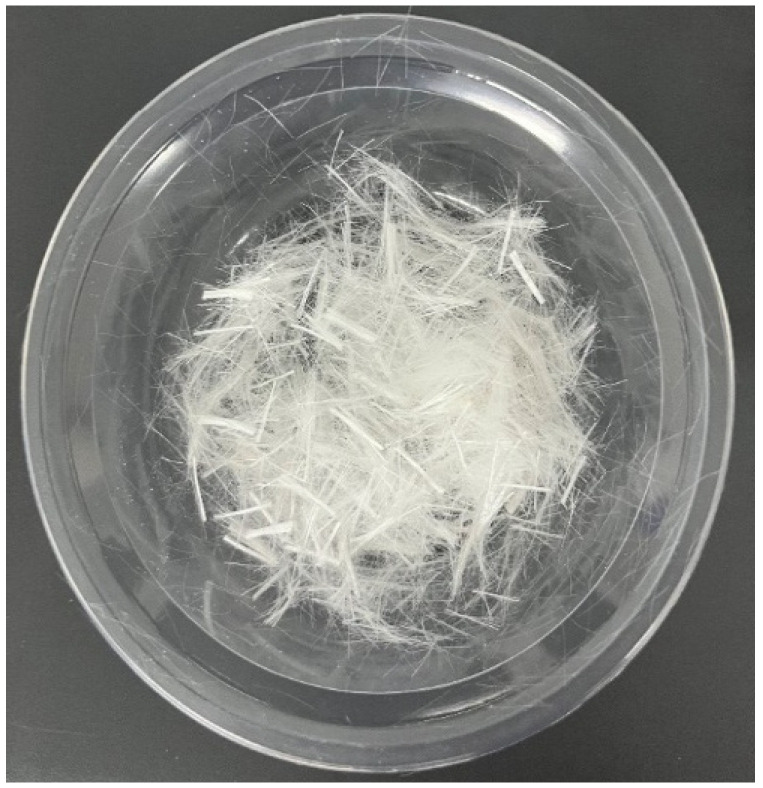
PVA fibers.

**Figure 2:**
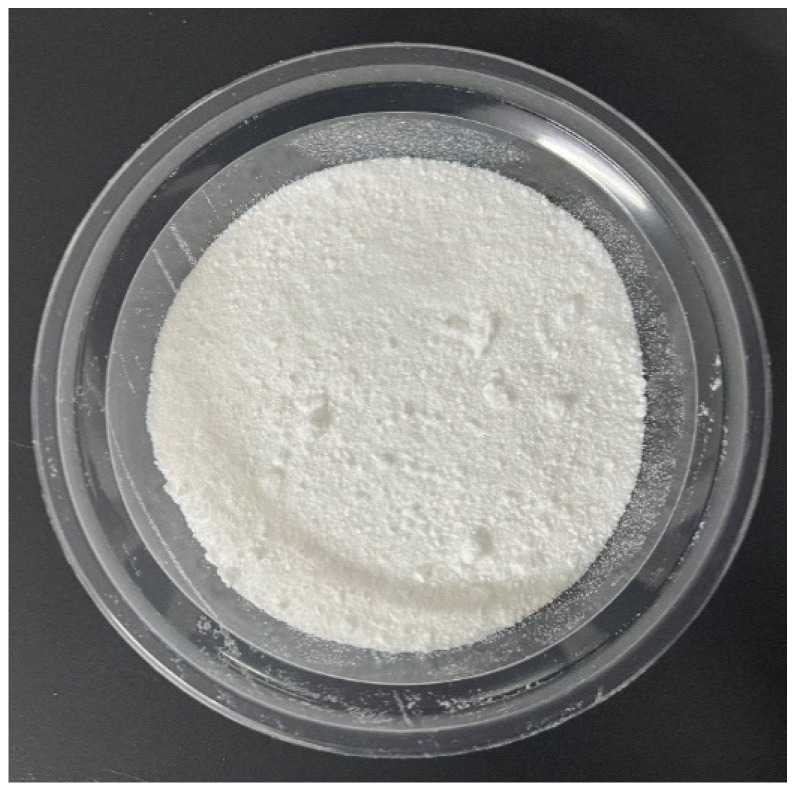
NS powder.

Ordinary PO 42.5 Portland cement produced by Harbin Yatai Co., Ltd. in Harbin, China with a density of 3.1 g/cm^3^ was used in this study; its components are listed in Table 1. The fine aggregate was made of natural quartz sand with a particle size of 350–500 µm, and the coarse aggregate consisted of continuously graded gravel with a particle size of 10–31.5 mm. The water used for mixing was city tap water, and the water–cement ratio (W/C) was 0.32. The water-reducing agent was a polycarboxylic acid high-efficiency water-reducing agent, and the water-reducing rate was 25%. The fibers used in this test were K-II type PVA fibers produced by Kuraray Company of Chiyoda City, Japan; their appearance is shown in Figure 1, and the mechanical properties of the fibers are listed in Table 2. The NS was the VKSH-30 type produced by Hangzhou Wanjing New Material Co. Ltd. (Hangzhou, China), shown in Figure 2, with the mechanical properties listed in Table 3.

### 2.2. Mix Proportions and Specimen Preparation

A total of 16 groups of cementitious composite specimens were designed to be tested in this experiment, including a control group (C0; without PVA fibers and NS), PVA fiber-reinforced cementitious composites (PFRCCs) containing only different proportions of PVA fibers (P-0.1 to P-0.9), and a series of NPFRCC groups containing different proportions of PVA fibers and NS (PN-0.3-0.5 to PN-0.5-2.5) in the NPFRCC group. Fixed values were used for quartz sand, water, coarse aggregate, and water-reducing for each experimental group, which were 703 kg/m^3^, 157 kg/m^3,^ 1100 kg/m^3^, and 8.3 kg/m^3^, respectively. The specific mixing ratios are shown in Table 4, where C0 stands for the control group, P for the PVA fiber alone specimen group, and PN for the compounded PVA fiber and NS specimen group. For example, P-0.3 represents the specimen group with 0.3% fiber doping alone, and PN-0.3-0.5 represents the specimen group with 0.3% PVA fiber and 0.5% NS doping.

The fresh cementitious composites were mixed using a laboratory Hobart mixer as per the Chinese GB/T 50082-2009 specification [26]. The dry sand and cement were mixed for 1 min, half of the dry NS and half of the dry PVA fiber quantities were added in two portions and mixed for 1 min, then the dry coarse aggregate and remaining NS and PVA fiber quantities were added in two portions and mixed dry for 1 min, and finally, the weighed water and additives were added to the mixture and mixed for 2 min. The mixture was subsequently poured into the specimen mold, which was placed on a shaking table for 24 h to ensure densification, after which it was stripped from the mold and placed into a standard curing box at a temperature of 20 ± 2 °C and relative humidity of 95% for 24 d. The specimen was subsequently removed and submerged in a 3.5% NaCl solution for 4 d, where it reached a final age of 28 d prior to testing.

### 2.3. Test Methods

#### 2.3.1. Saline Freeze–Thaw Cycle Tests

**Figure 3:**
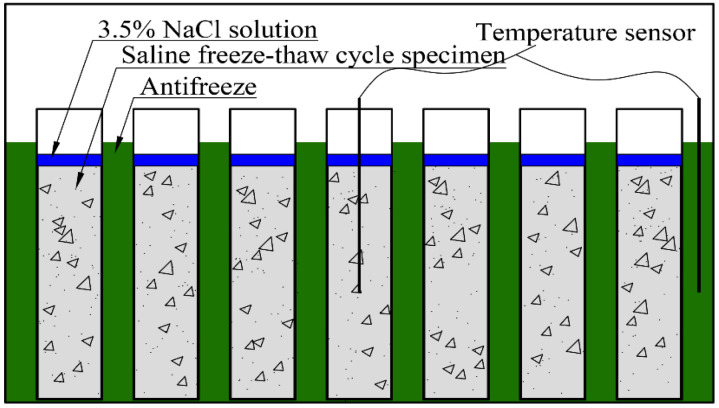
Concrete rapid freeze–thaw test setup.

The saline freeze–thaw cycle tests were conducted with reference to the rapid freeze–thaw method in the Chinese GB/T 50082-2009 specification [26]. The test specimens were 100 mm × 100 mm × 400 mm prismatic specimens, h=t=100mm, L=400mm, and each group of specimens totaled three. After the specimens were maintained for 24 d, the specimens were immersed in a NaCl solution with a concentration of 3.5% for 4 d, and after reaching the age of 28 d, each specimen was taken out of the NaCl solution, the moisture on the surface was wiped off, and then its initial mass and dynamic elastic modulus were measured. Next, the specimens were placed into a TYC-HDK rapid freeze–thaw concrete testing machine, shown in Figure 3, to carry out the freeze–thaw cycle test (in which each cycle was 4 h long) over a temperature range of −18 ± 2 °C to 5 ± 2 °C. Each specimen was removed from the machine every 25 cycles, its surface moisture was wiped off, and its transverse fundamental frequency and mass was measured.

The relative dynamic modulus of elasticity $P_{ni}$ (%) of specimen *i* after *n* freeze–thaw cycles calculated from reference [26], accurate to the tenths, was calculated as follows:

(1) $$P_{ni}=\frac{f_{ni}^{2}}{f_{0i}^{2}}\times 100$$

where $f_{ni}$ denotes the transverse fundamental frequency (Hz) of the *i*th concrete specimen after *n* freeze–thaw cycles and $f_{0i}$ denotes the transverse fundamental frequency (Hz) of the *i*th concrete specimen before any freeze–thaw cycles. The average $P_{ni}$ from three specimens was reported as $P_{n}$. Generally, a specimen was considered to be damaged after freeze–thaw cycles if the $P_{ni}$ was below 60%.

The mass loss rate $W_{ni}$ (%) of specimen *i* after *n* freeze–thaw cycles, accurate to the hundredths, was calculated as follows:

(2) $$W_{ni}=\frac{G_{0i}-G_{ni}}{G_{0i}}\times 100$$

where $G_{0i}$ represents the mass (kg) of the *i*th concrete specimen before freeze–thaw cycles and $G_{ni}$ represents the mass (kg) of the *i*th concrete specimen after *n* freeze–thaw cycles. The average $W_{ni}$ from three specimens was reported as $W_{n}$. Generally, if the $W_{ni}$ was greater than 5%, the specimen was considered to have been destroyed after freeze–thaw cycles.

#### 2.3.2. Compressive Strength Tests

Concrete cube compressive strength tests were carried out on NPFRCC specimens after 0, 25, 50, 75, and 100 salt freeze–thaw cycles according to the Chinese standard GB/T 50081-2019 [27], with three in each group and dimensions of 100 mm × 100 mm × 100 mm, using the TYA-3000E microcomputer-controlled constant loading compression tester from WuxiXinluda Instruments Co. Ltd. (Wuxi, China), and the compressive strengths were calculated using the method specified in specification [27], using Equation (3):

(3) $$f_{c}=\frac{F_{cc}}{A_{cc}}$$

where $f_{c}$ denotes the compressive strength of the cubic specimen (MPa), $F_{cc}$ denotes the destructive load of the specimen (N), and $A_{cc}$ denotes the pressure-bearing area of the specimen (mm^2^). The calculation results were multiplied by a 0.95 conversion factor and the average value of three tests was reported as the representative compressive strength. Generally, a specimen after a freeze–thaw cycle was considered to be completely destroyed if the $f_{c}$ was reduced to less than 75% of the initial value.

#### 2.3.3. Fracture Toughness

**Figure 4:**
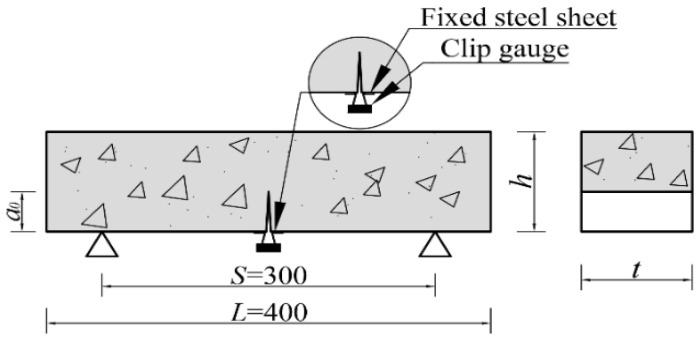
Fracture test specimen size diagram (units: mm).

This study applied the three-point bending beam method to analyze the specimens. The Chinese specification DL/T 5332-2005 [28] was adopted. To evaluate fracture toughness, a 3 ± 1 mm wide slit was cut across the midspan of each specimen to a depth $a_{0}=40\;mm,$ with a slit depth-to-specimen height ratio of $a_{0}/h=0.4$. The three-point bending test was subsequently conducted using an electro-hydraulic servo universal testing machine, with a span of *S* = 300 mm between the two supports, and a span-to-height ratio of S/h=3.0. The loading process was displacement-controlled to maintain a loading rate of 0.06 mm/min. A clip-type extensometer with a range of 5 mm and an accuracy of 0.5 mm was installed across the slit at the bottom of each specimen to accurately measure the crack mouth opening displacement (*CMOD*). The fracture test specimen size diagram is shown in Figure 4. The fracture toughness and fracture energy of each specimen were calculated from its resulting *P–CMOD* curves as the average value of three test results.

**Figure 5:**
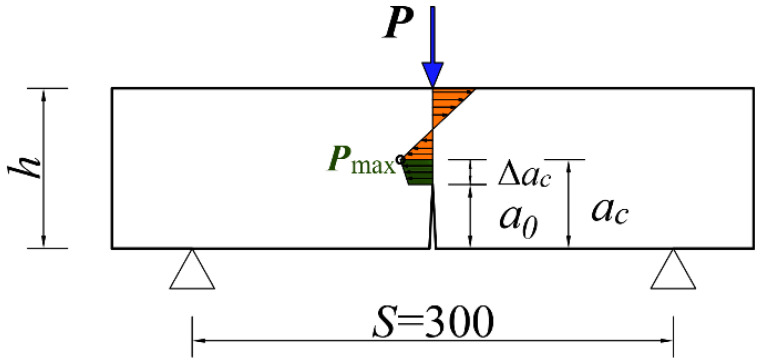
Stress distribution in fractured specimens (units: mm).

The double-K fracture theory (DKFT) has gradually matured through in-depth research to demonstrate a simple and clear result. Furthermore, it employs a straightforward testing process that yields results without requiring cumbersome numerical calculations, reducing the data analysis workload. Therefore, it has been adopted by many researchers for the analysis of three-point bending beam test results. The calculations of the initiation toughness, the destabilization toughness, and the fracture energy, i.e., Equations (4)–(10), in this paper were based on references [28]. The stress distribution of the specimen under peak load is shown in Figure 5. Using the DKFT, when the external load reached its peak value ($F_{\max}$), *CMOD* reached its critical value, and the crack length became the critical effective crack length ($a_{c}$), which can be calculated according to DL/T 5332-2005 [28] as follows:

(4) $$a_{c}=\frac{2}{\pi }\left(h+h_{0}\right)\arctan\sqrt{\frac{tEV_{c}}{32.6F_{\max}}-0.1135}-h_{0}$$

where $h_{0}$ denotes the thickness of the steel plate at the knife edge of the device-clamped extensometer (0.001 m), t denotes the thickness of the specimen (0.1 m), $V_{c}$ denotes the critical value of *CMOD* measured by the clamped extensometer (µm), $F_{\max}$ denotes the peak load (kN), and E denotes the modulus of elasticity (GPa), which can be calculated by the following equation:

(5) $$E=\frac{1}{tc_{i}}\left[3.70+32.60\tan^{2}\left(\frac{\pi }{2}\frac{a_{0}+h_{0}}{h+h_{0}}\right)\right]$$

where $c_{i}$ denotes the ratio of *CMOD* to load in the linear stage, μm/kN.

The crack development process can be divided into three stages as the applied load increases: crack initiation, stable crack extension, and destabilizing damage. The value of $K_{IC}^{ini}$ describes the crack initiation toughness ($MPa\cdot m^{1/2}$), representing the stress intensity factor at the tip of the initial crack after the externally applied load has reached the crack initiation load, and can be obtained by the following equation:

(6) $$K_{IC}^{ini}=\frac{1.5\left(F_{ini}+\frac{mg}{2}\times 10^{-2}\right)\times 10^{-3}\times S\times \sqrt{a_{0}}}{th^{2}}f\left(\lambda_{0}\right)$$

where m denotes the mass of the specimen between supports (kg), which is converted from the total mass of the specimen in accordance with S/L; $F_{ini}$ denotes the crack initiation load; g denotes the acceleration due to gravity ($9.81m/s^{2}$); S denotes the span (m) between the two supports; and $f\left(\lambda_{0}\right)$ is calculated as follows:

(7) $$f\left(\lambda_{0}\right)=\frac{1.99-\lambda_{0}\left(1-\lambda_{0}\right)\left(2.15-3.93\lambda_{0}+2.7\lambda_{0}{\;\;}^{2}\right)}{\left(1+2\lambda_{0}\right){\left(1-\lambda_{0}\right)}^{3/2}}$$

where $\lambda_{0}=a_{0}/h$.

Next, the value of $K_{IC}^{un}$ describes the destabilizing toughness ($MPa\cdot m^{1/2}$), representing the stress intensity factor at the critical effective crack tip under the peak external load, and is calculated as follows:

(8) $$K_{IC}^{un}=\frac{1.5\left(F_{\max}+\frac{mg}{2}\times 10^{-2}\right)\times 10^{-3}\times S\times \sqrt{a_{c}}}{th^{2}}f\left(\lambda_{c}\right)$$

where $f\left(\lambda_{c}\right)$ is calculated by the following equation:

(9) $$f\left(\lambda_{c}\right)=\frac{1.99-\lambda_{c}\left(1-\lambda_{c}\right)\left(2.15-3.93\lambda_{c}+2.7\lambda_{c}{\;\;}^{2}\right)}{\left(1+2\lambda_{c}\right){\left(1-\lambda_{c}\right)}^{3/2}}$$

where $\lambda_{c}=a_{c}/h$.

#### 2.3.4. Fracture Energy $G_{F}$

The energy expended in the direction of the crack opening when fracture damage occurs in the material is given by $G_{F}$. It reflects the ability of the material to resist the crack destabilization and is a key index for analyzing the fracture process of any material; it can be calculated by the following equation:

(10) $$G_{F}=\frac{W_{0}}{t\left(h-a_{0}\right)}+\frac{mg\delta_{0}}{t\left(h-a_{0}\right)}$$

where $W_{0}$ corresponds to the area under the *P–CMOD* curve and $\delta_{0}$ indicates the maximum deflection.

## 3. Results and Discussion

### 3.1. Saline Freeze–Thaw Cycle Tests

#### 3.1.1. Apparent Morphology

**Figure 6:**
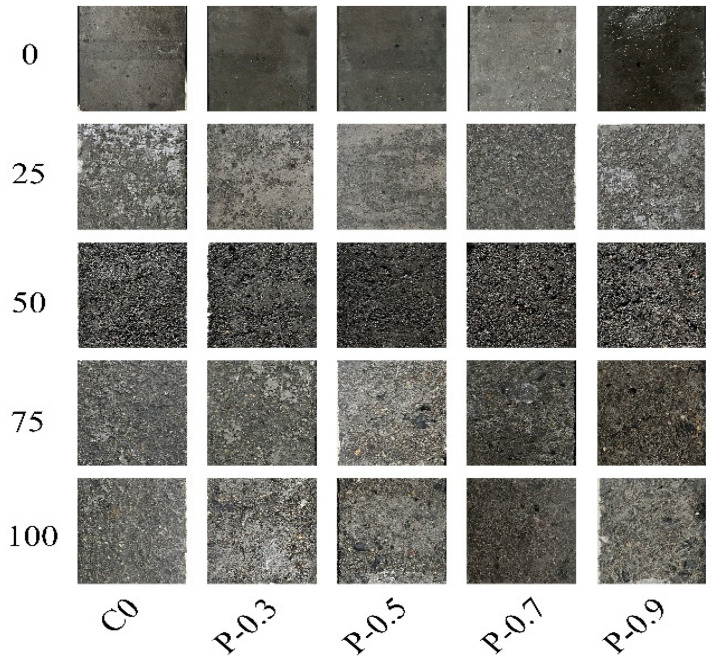
Surface morphologies of PFRCC specimens after saline freeze–thaw cycling.

**Figure 7:**
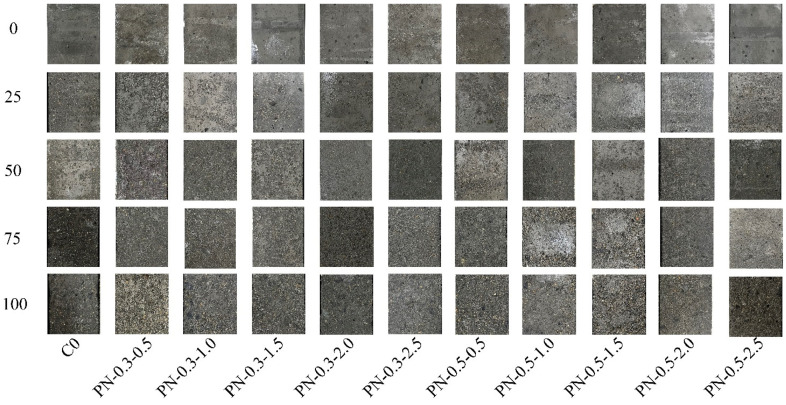
Surface morphologies of NPFRCC specimens after saline freeze–thaw cycling.

Concrete subjected to freeze–thaw cycling typically exhibits surface spalling and matrix cracking. The apparent morphologies of the specimens considered in this study clearly changed over 25, 50, 75, and 100 saline freeze–thaw cycles according to the quantities of NS and PVA fibers in their mixes, as shown in Figure 6 and Figure 7. The surface damage on each specimen gradually became more severe as the number of applied saline freeze–thaw cycles increased. At the beginning of the freeze–thaw cycling, the PFRCC specimens exhibited a gradual decrease in surface damage, with an increase in fiber content. Notably, when the fiber content was greater than 0.7%, the composite cementitious material was difficult to mix uniformly as the fibers were prone to agglomeration; the original porosity of the specimen increased accordingly, causing a large number of holes to appear on the specimen surface. After 100 freeze–thaw cycles, the aggregate at the corners of this specimen had detached, significantly reducing its freezing resistance.

An increase in NS doping effectively improved the freezing resistance of the NPFRCC specimens, reducing the apparent degree of damage owed to freeze–thaw cycling. Given the same fiber content, the surface damage to the specimens gradually decreased as the NS content increased. After 25 freeze–thaw cycles, the surface of each specimen exhibited different degrees of damage: the surface of the C0 was quite rough, whereas only a small quantity of cementitious material particles was dislodged from specimens containing NS and PVA. After 50 freeze–thaw cycles, the surface of the C0 was peeled off across a large area and small portions of fine aggregate were exposed, whereas specimens containing NS and PVA fibers remained in significantly better condition. Given the same fiber content, an increase in NS content caused the surface layer peeling to gradually weaken, and no exposed aggregates were observed. After 75 freeze–thaw cycles, the surface material of the C0 had peeled off across a large area of deep damage, with several holes appearing. Specimens with 0.3% fiber content exhibited partially exposed fine aggregate, with less exposure for specimens with 0.5% fiber content, though the quantity of holes in the matrix increased in both cases. After 100 freeze–thaw cycles, the surface of the C0 exhibited serious spalling of cementitious material, exposed coarse aggregate, and missing edges, and the structure of the entire specimen was quite loose. The exposed area of fine aggregates on the surfaces of the specimens with 0.3% fiber content increased, and the degree of this damage gradually decreased with increasing NS content. The surfaces of the specimens with 0.5% fiber content became rough, owing to the small quantity of free water in the cementitious material during the mixing process as well as the fiber agglomeration phenomenon, in which the fiber adheres to the surface of the specimen, resulting in holes on the surface.

The surface state of each specimen demonstrated that after freeze–thaw cycling, NPFRCC specimens exhibit damage states such as spalling of cementitious materials, exposed aggregates, and missing edges. This damage process occurred from the outside to the inside and gradually became serious. The observed changes in surface state indicate that both NS and PVA fibers can improve the frost resistance of concrete, with the enhancement provided by NS superior to that provided by PVA fibers alone.

#### 3.1.2. Mass Loss Rate

**Figure 8:**
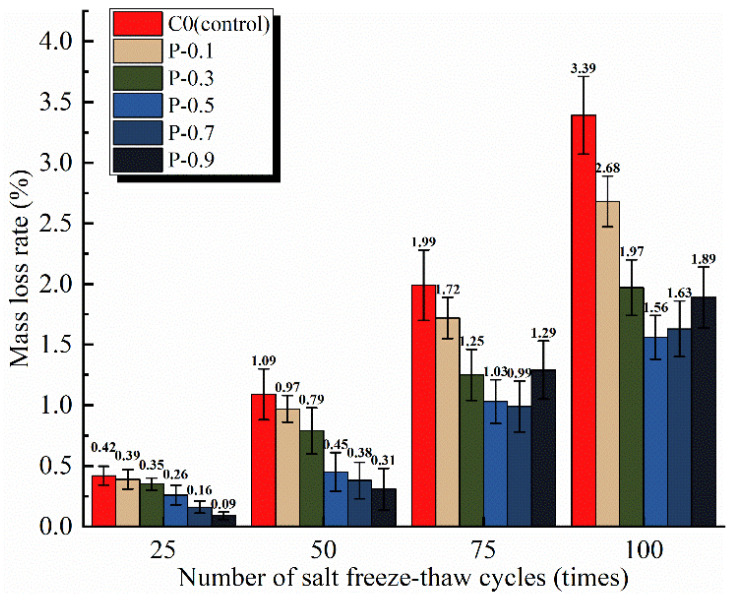
Mass loss rate of PFRCC specimens according to applied saline freeze–thaw cycles.

**Figure 9:**
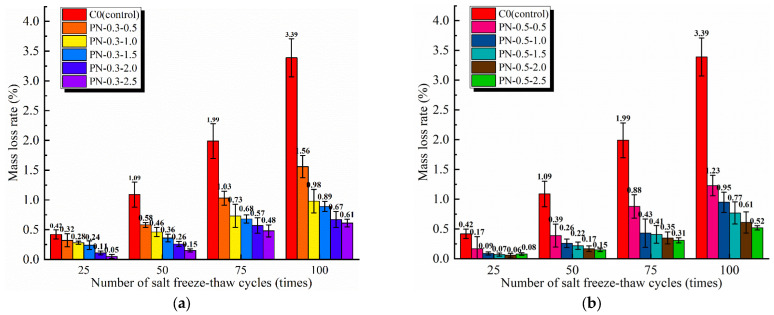
Mass loss rate of PFRCC specimens according to applied saline freeze–thaw cycles: (**a**) 0.3% fiber content; (**b**) 0.5% fiber content.

The mass loss rate is an indispensable index for measuring the freezing resistance of concrete; the smaller the mass loss of the specimen after several freeze–thaw cycles, the better its freezing resistance. The mass loss rates of the PFRCC and NPFRCC specimens after different numbers of freeze–thaw cycles are shown in Figure 8 and Figure 9, respectively. The figures indicate that the mass loss of the matrix increased gradually with increasing freeze–thaw cycles. This occurred because the holes in each specimen provided a channel for the expansion and contraction of the saline solution, and the subsequent enlargement of tiny cracks allowed more water molecules to enter the interior of the specimen, aggravating the damage.

A relevant body of literature [23,24,29] has analyzed the flow, mechanical properties, and porosity of cementitious composites and concluded that fiber incorporation in cementitious composites should be less than 1%. Because fiber content has a significant impact on the various properties of concrete, the fiber content considered in this study was varied in 0.2% increments between 0.1% and 0.9% to compare the performance deterioration of the specimens after 100 saline freeze–thaw cycles, as shown in Figure 8. Clearly, as the fiber content increased, the interior of the matrix became denser. During freeze–thaw cycles, the fibers mixed in the specimen interior began to bridge potential cracks, resisting the freezing force. The effect of this resistance was significant, with the rate of mass loss noticeably decreasing with increasing fiber content. Indeed, the mass loss rates for specimens P-0.1, P-0.3, P-0.5, P-0.7, and P-0.9 were 11.01%, 27.52%, 58.72%, 65.14%, and 71.56% smaller, respectively, than that of the control specimen, with no fibers after 25 freeze–thaw cycles. After 100 freeze–thaw cycles, the mass loss of each group of fiber-alone doped specimens was reduced by 20.84%, 41.81%, 53.92%, 51.85%, and 44.17%, respectively, compared to the fiber-free control specimens. Thus, once the fiber content exceeded 0.5%, there was slower improvement in the mass loss rate, suggesting that while an appropriate fiber content can help to improve freezing resistance, too many fibers can lead to fiber agglomeration, forming cavities as well as numerous tiny cracks inside the matrix that increase available sites for damage.

The effect of coupling between the NS and PVA fibers on the fracture properties of the specimens subjected to saline freeze–thaw cycles was considered for fiber contents of 0.3% and 0.5% and NS contents of 0.5%, 1.0%, 1.5%, 2.0%, and 2.5%. After 100 freeze–thaw cycles, the results indicate that the incorporation of NS effectively promoted cement hydration while simultaneously filling the pore structure. Therefore, early in the freeze–thaw cycling, damage primarily occurred on the specimen surfaces. The mass losses of NPFRCC specimens with different NS and fiber contents are shown in Figure 9; an increase in NS content, given the same fiber content, caused the mass loss of the specimens to gradually decrease. The specimens with 0.5% fiber content clearly exhibited smaller mass losses of 0.06–0.42% after 25 freeze–thaw cycles because of their denser matrices. After 50 freeze–thaw cycles, the freeze–thaw damage developed from the surfaces into the interiors of the specimens, and the mass loss decreased significantly with increasing fiber contents. After 100 freeze–thaw cycles, the tiny pores inside each specimen matrix were continuously penetrated by the saline solution, contributing to the development of cracks, and making the material fragile. The mass loss rates from specimens PN-0.3-0.5, PN-0.3-1.0, PN-0.3-1.5, PN-0.3-2.0, and PN-0.3-2.5 were 53.98%, 71.09%, 73.75%, 80.24%, and 82.01% smaller, respectively, than that of the C0; the mass loss rates from specimens PN-0.5-0.5, PN-0.5-1.0, PN-0.5-1.5, PN-0.5-2.0, and PN-0.5-2.5 were 63.72%, 71.98%, 77.29%, 82.01%, and 84.66% smaller, respectively, than that of the C0. Clearly, the bridging effect of the fibers and the filling effect of NS in the NPFRCC specimens effectively inhibited the spalling of its matrix and aggregate.

#### 3.1.3. Relative Dynamic Modulus of Elasticity

**Figure 10:**
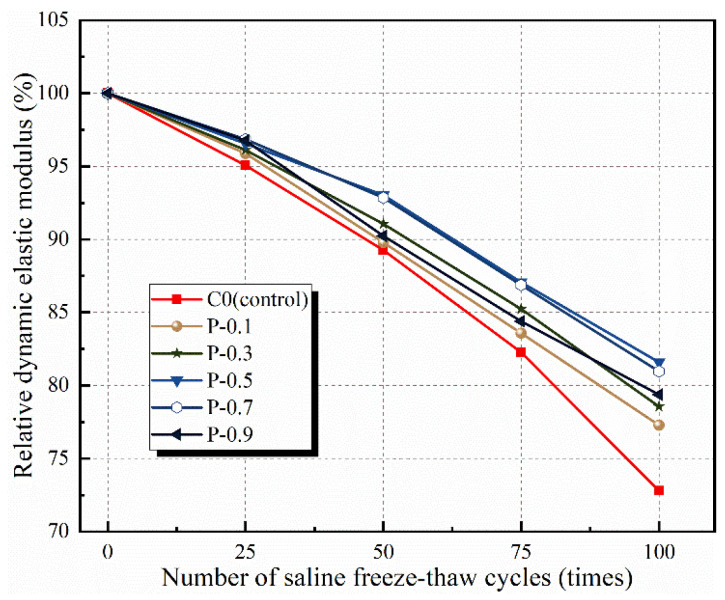
Relative dynamic modulus of elasticity of PFRCC specimens according to applied saline freeze–thaw cycles.

**Figure 11:**
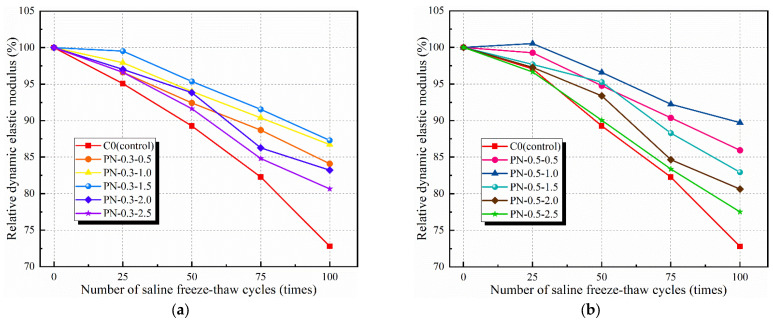
Relative dynamic modulus of elasticity of NPFRCC specimens according to applied saline freeze–thaw cycles: (**a**) 0.3% fiber content; (**b**) 0.5% fiber content.

The relative dynamic elastic moduli of the PFRCC and NPFRCC specimens are shown in Figure 10 and Figure 11, respectively, according to the number of applied saline freeze–thaw cycles. The relative dynamic elastic modulus for each specimen series decreased gradually with increasing applied freeze–thaw cycles, with the most serious damage observed for the C0. Regardless of whether the PVA fibers were provided alone or mixed with NS, the relative dynamic elastic modulus decayed slowly before 50 freeze–thaw cycles and accelerated thereafter, but all values were consistently smaller than that of the C0. Duan et al. [5] also found in the course of their study that 50 cycles was the cut-off point using chloride and sulfate as the freeze–thaw cycling media. A comparison of the test results shows that NS incorporation can effectively mitigate freeze–thaw damage, but the overall trend is the same.

Figure 10 shows that for the PFRCC specimens, the relative dynamic elastic modulus did not increase with PVA fiber contents, the bridging effect was not obvious, and the presence of too many fibers in the mix made them prone to agglomeration, promoting the formation of voids in the matrix body. Simultaneously, the increased fiber content and matrix interface transition zone material increased the number of primary cracks observed after several freeze–thaw cycles, causing serious internal damage. Indeed, after 100 freeze–thaw cycles, the dynamic elastic moduli of P-0.1, P-0.3, P-0.5, P-0.7, and P-0.9 increased by 10.77%, 16.28%, 29.33%, 26.62%, and 19.77%, respectively, compared with that of the C0.

Figure 11 shows that PN-0.5-1.0 exhibited a higher dynamic modulus of elasticity after 25 freeze–thaw cycles because the incompletely hydrated cement within it still absorbed water and continued to hydrate at the beginning of freeze–thaw cycling, filling the pores with hydration products and densifying the internal structure. This phenomenon was also verified by Xu et al. [30]. NS with PVA fibers can effectively attenuate freeze–thaw damage to the interior of the matrix to a certain extent. After 100 freeze–thaw cycles, the relative dynamic elastic moduli of PN-0.3-0.5, PN-0.3-1.0, PN-0.3-1.5, PN-0.3-2.0, and PN-0.3-2.5 decreased to 84.09%, 86.71%, 87.29%, 83.22%, and 80.65%, respectively, for their initial values, with the value for PN-0.3-1.5 representing a 53.31% improvement over that of the C0 at the time; the relative dynamic elastic moduli of PN-0.5-0.5, PN-0.5-1.0, PN-0.5-1.5, PN-0.5-2.0, and PN-0.5-2.5 decreased to 85.94%, 89.72%, 82.93%, 80.63%, and 77.52%, respectively, for their initial values, with the value for PN-0.5-1.0 representing a 62.23% improvement over that of the C0 at the time. Although the relative dynamic elastic modulus at the time of specimen destruction remained greater than 60%, particular fiber and NS content ranges can still be recommended: the saline freeze–thaw resistance of the composite cementitious material was better when the NS content was 1.0–1.5% and the PVA fiber content was 0.3–0.5%.

The test results show that both NS and PVA fibers improved the frost resistance of concrete, and the filling effect of NS on the concrete matrix and the bridging effect of the fibers effectively inhibited the spalling of concrete during freeze–thaw cycles, but the comparative analysis reveals that the effect of NS on the rate of mass loss of concrete was greater than that of PVA fibers. When the NS dosage exceeded 1.5%, a large amount of free water was consumed during the hydration process, which led to more internal original damage, and the relative dynamic elastic modulus of the specimens after 50 freeze–thaw cycles all decreased by a larger amount.

### 3.2. Compressive Strength

**Figure 12:**
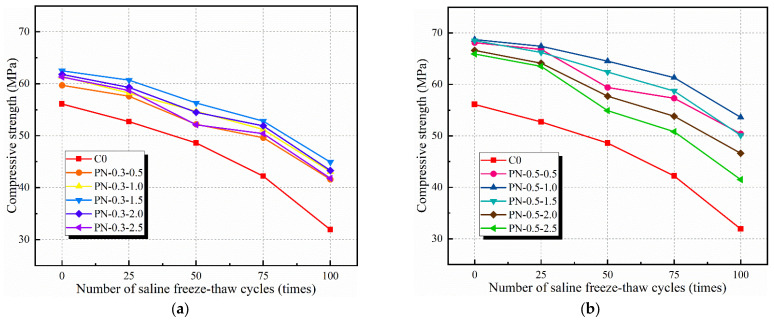
Compressive strength of NPFRCC specimens under different number of saline freeze–thaw cycles: (**a**) 0.3% fiber content; (**b**) 0.5% fiber content.

The cubic compressive strengths of each specimen according to the number of applied freeze–thaw cycles are shown in Figure 12. When the specimen fiber content was 0.3%, there was sufficient free water to fully react with NS to produce C-H-S gel. Thus, the compressive strengths of specimens PN-0.3-0.5, PN-0.3-1.0, PN-0.3-1.5, PN-0.3-2.0, and PN-0.3-2.5 increased by 6.03%, 8.63%, 10.24%, 9.22%, and 8.48%, respectively, over that of the C0. Owing to the large specific surface area of NS, once the NS content exceeded 2%, a large quantity of free water had been consumed, increasing the difficulty of consolidating the material and thereby increasing the number of pores. The results of Ling’s study also showed that with too much NS admixture, the specific surface area of the material is too large and the cement is difficult to hydrate, making the concrete’s strength lower [31]. After 100 freeze–thaw cycles, the compressive strengths of specimens PN-0.3-0.5, PN-0.3-1.0, PN-0.3-1.5, PN-0.3-2.0, and PN-0.3-2.5 were 48.4%, 50.17%, 59.93%, 54.35%, and 29.27% higher, respectively, than that of the C0.

The compressive strengths of PN-0.5-0.5, PN-0.5-1.0, PN-0.5-1.5, PN-0.5-2.0, and PN-0.5-2.5 were 19.61%, 24.29%, 22.11%, 16.93%, and 13.91% larger, respectively, than that of C0. Thus, for a fiber content of 0.5% and NS content of less than 1%, the density of the NS and the bridging provided by the fibers were excellent; however, when the NS content was greater than 1%, the cement could not fully undergo hydration owing to a lack of free water. Combined with the phenomenon of fiber aggregation, this caused the strength of the concrete to decrease and the volume of internal pores to increase. Therefore, after several salt freeze–thaw cycles, the matrix microcracking increased, the saline solution entered the matrix and began to corrode it, and the degree of damage increased with the ongoing expansion and contraction of water. After 100 freeze–thaw cycles, the compressive strengths of PN-0.5-0.5, PN-0.5-1.0, PN-0.5-1.5, PN-0.5-2.0, and PN-0.5-2.5 were 69.33%, 80.14%, 65.51%, 34.49%, and 18.11% larger, respectively, that that of the C0. Note that PN-0.5-2.5 did not exhibit a significant increase in compressive strength, owing to the heavy damage it suffered from the freeze–thaw cycling. After 100 saline freeze–thaw cycles, only the PN-0.5-1.0 group complied with the standard requirements, and the compressive strength of the control group, C0, had the largest decrease of 43.14%.

The above test results show that PVA fibers can significantly enhance the compressive strength of concrete cubes. After the specimen is subjected to the freeze–thaw cycle, NaCl in the salt solution reacts with the concrete products to generate CaCl_2_ with higher solubility, which exacerbates the destruction of the concrete specimen by the salt–freeze cycle. At the same time, the saturation of the salt solution that penetrates into the interior of the matrix grows faster, and the infiltration and icing pressure are higher, which leads to a rapid decrease in the mechanical properties of the specimen after freeze–thaw. When the dosage of NS is low (dosage less than 1.5%), the incorporation of NS with PVA fibers significantly enhances compressive strength, which slowly decreases with the increase in the number of freezing and thawing cycles; when the dosage of NS is more than 2%, the primary pores inside the matrix cause the concrete freeze–thawing damage to intensify, and the frost-resistance performance decreases significantly.

### 3.3. Fracture Toughness

**Figure 13:**
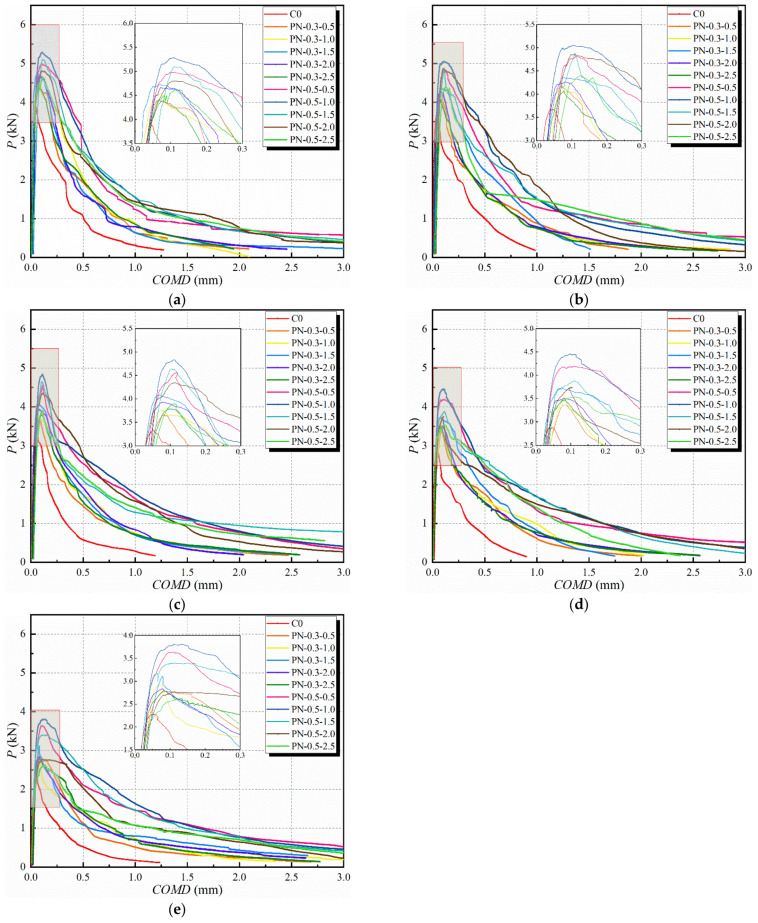
*P–COMD* curves for NPFRCC specimens according to applied saline freeze–thaw cycles: (**a**) 0 cycles; (**b**) 25 cycles; (**c**) 50 cycles; (**d**) 75 cycles; and (**e**) 100 cycles.

Figure 13 shows the *P–COMD* curves of the NPFRCC specimens after 0, 25, 50, 75, and 100 saline freeze–thaw cycles, indicating that the location of the peak load did not change significantly with NS content at a given fiber content. Although NS promoted the hydration reaction to generate C-S-H gels that filled the matrix pores and formed a three-dimensional network structure, it primarily improved the degree of densification and the microstructure of the composites and, as such, had a limited effect on the improvement of their fracture properties. The bridging effect provided by PVA fibers had a significant effect on the fracture properties of the composites. Indeed, freeze–thaw cycling increased the destabilizing load of PN-0.5-0.5 and PN-0.5-1.0 by 13.5% and 16.7% compared to that of PN-0.3-0.5 and PN-0.3-1.0, respectively. Thus, the specimens with 0.5% fiber content exhibited significantly better toughness than those with 0.3% fiber content.

As the number of applied freeze–thaw cycles increased, the initiation load and destabilizing load for each specimen type exhibited different degrees of attenuation, and the attenuation of fracture toughness matched that of the compressive strength. The fracture toughness attenuation was slow before 50 freeze–thaw cycles, then accelerated. At the beginning of freeze–thaw cycling, there were no obvious cracks in the middles or outsides of the specimens and the fracture toughness primarily depended on the cementitious material, aggregate, and fiber content. Notably, after 100 freeze–thaw cycles, the destabilizing loads of P-N-0.3-1.0, P-N-0.3-1.5, P-N-0.5-0.5, and P-N-0.5-1.0 were 28.16%, 36.87%, 67.80%, and 75.59% larger, respectively, than that of the C0.

**Figure 14:**
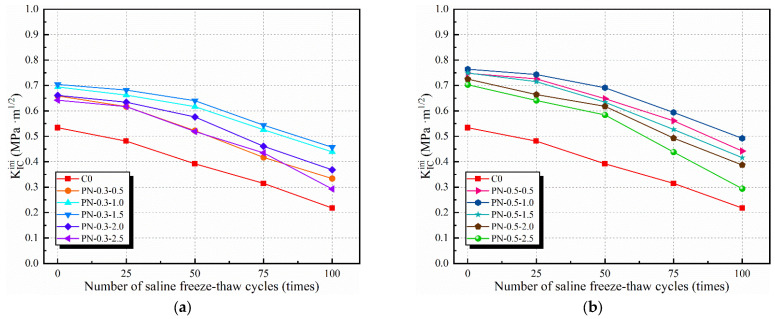
$K_{IC}^{ini}$ of NPFRCC specimens according to applied saline freeze–thaw cycles: (**a**) 0.3% fiber content; (**b**) 0.5% fiber content.

**Figure 15:**
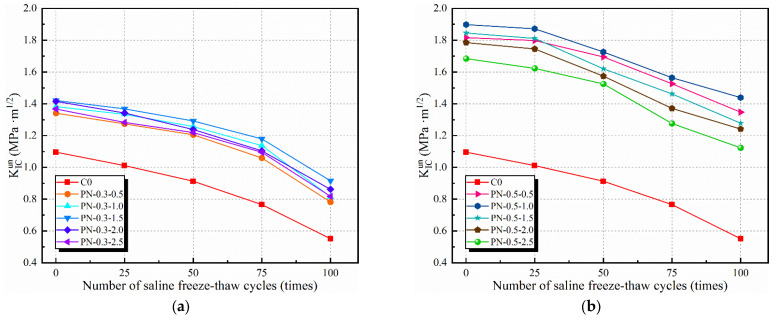
$K_{IC}^{un}$ of NPFRCC specimens according to applied saline freeze–thaw cycles: (**a**) 0.3% fiber content; (**b**) 0.5% fiber content.

Based on the DKFT and Equations (4)–(9), the effect of the number of saline freeze–thaw cycles on the $K_{IC}^{ini}$ and $K_{IC}^{un}$ of an NPFRCC specimen can be derived according to the NS and fiber contents. Figure 14 and Figure 15 show that fiber content played a significant role in the fracture toughness of the specimens. Furthermore, when the fiber content was fixed, there were differences according to the NS content, owing to the fact that the C-S-H gel generated by NS not only optimized the pore structure of the matrix but also improved the ITZ properties, as well as the adhesion of the PVA fibers. Before freeze–thaw cycling, the $K_{IC}^{ini}$ values for specimens P-N-0.3-1.0, P-N-0.3-1.5, P-N-0.5-0.5, and P-N-0.5-1.0 (which exhibited better performance) were 29.96%, 31.84%, 40.07%, and 43.07% higher, respectively, than that of the C0, and their $K_{IC}^{un}$ values were 26.09%, 29.65%, 65.69%, and 73.18% higher, respectively. After 25 freeze–thaw cycles, there was a slight decrease in fracture toughness with ongoing internal damage and the enhancement of freeze–thaw resistance by the inclusion of NS and PVA fibers gradually became more obvious. After 100 freeze–thaw cycles, specimens P-N-0.3-1.0, P-N-0.3-1.5, P-N-0.5-0.5, and P-N-0.5-1.0 exhibited $K_{IC}^{ini}$ values that were 101.38%, 109.63%, 102.75%, and 125.69% higher, respectively, than that of the C0, and $K_{IC}^{un}$ values that were 47.91%, 66.06%, 144.46%, and 161.16% higher, respectively.

As can be seen from the test results, under the same number of freeze–thaw cycles, the starting cracking toughness and destabilizing toughness of the specimen doped with NS and PVA fibers are significantly larger than that of the control group, C0, indicating that NS and PVA fibers have a significant inhibitory effect on the destabilizing damage of the specimen, and the enhancement of the starting cracking toughness and destabilizing toughness of the specimen doped with NS and PVA fibers shows a trend of increasing first and then decreasing with the increase in the doping amount of the materials. The reason is that the appropriate amount of NS can effectively enhance the hydration reaction rate to generate C-S-H to make the concrete structure dense, so that the freeze–thaw cycle on the specimen caused less damage, while too much NS (wt% > 1.5%) doping led to an increase in original pores in the specimen, and the damage increased in the late freeze–thaw cycle. The PVA fiber itself was almost unaffected by the freeze–thaw cycle, so after many freeze–thaw cycles, the bridging effect of the PVA fiber was not lost, while the higher doping of the fiber intricately distributed in the crack expansion path could effectively resist fracture damage to the specimen.

### 3.4. Fracture Energy

**Figure 16:**
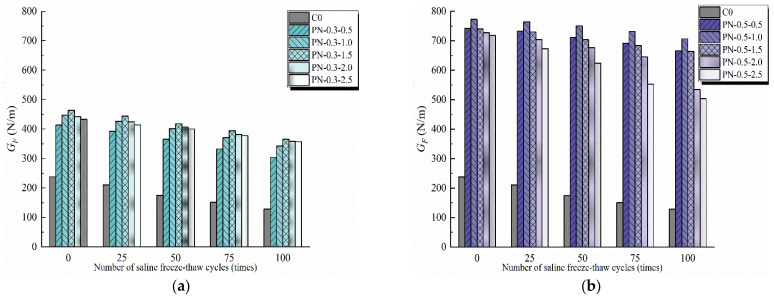
Fracture energy of NPFRCC specimens according to applied saline freeze–thaw cycles: (**a**) 0.3% fiber content; (**b**) 0.5% fiber content.

The fracture energy, quantified by $G_{F}$, is a vital parameter characterizing the difficulty of creating new cracks in concrete during the fracturing process. The fracture energy results obtained in this study indicate that the incorporation of NS and PVA fibers had a significant influence on damage to the specimens during freeze–thaw cycling. As shown in Figure 16, the bridging effect provided by the PVA fibers increased the toughness of the cementitious composite, increasing the energy required to continue the fracture process, as reflected by the significant increase in $G_{F}$ values. Note that the increase in $G_{F}$ with NS content, given the same fiber content, was less than that with fiber content, given the same NS content, because NS primarily prevents cracking at the microscopic scale.

Figure 16a,b show the change in the $G_{F}$ values of specimens with different fiber contents according to the number of applied freeze–thaw cycles. When the fiber content was fixed, $G_{F}$ exhibited a tendency to increase, then decrease as the NS content increased from 0.5% to 2.5%. Before freeze–thaw cycling, the $G_{F}$ values of specimens PN-0.3-0.5, PN-0.3-1.0, PN-0.3-1.5, PN-0.3-2.0, and PN-0.3-2.5 were 79.97%, 94.32%, 101.63%, 92.49%, and 88.19% higher, respectively, than that of the C0, and those of PN-0.5-0.5, PN-0.5-1.0, PN-0.5-1.5, PN-0.5-2.0, and PN-0.5-2.5 were 222.83%, 236.01%, 222.05%, 216.24%, and 212.01% higher, respectively. These results demonstrate that an appropriate quantity of NS promotes the hydration of cement to generate C-S-H, enhancing the adhesion between the matrix and fibers as well as between fibers themselves, thereby improving the freezing resistance of the structure.

However, the synergistic effect of NS and PVA fibers on $G_{F}$ differed with their respective contents. Figure 16 indicates that the effect of NS content on $G_{F}$ was significantly higher after 100 freeze–thaw cycles than before any cycles were applied. This occurred because the appropriate quantities of NS and PVA fibers can compensate for the internal defects in the matrix and reduce the degree of breakage within, owing to freeze–thaw cycling, whereas excessive NS can lead to the agglomeration of nanoparticles and fibers, resulting in a weak matrix with damage that is macroscopically manifested as a reduction in the fracture toughness and fracture energy. After 100 freeze–thaw cycles, PN-0.3-0.5, PN-0.3-1.0, PN-0.3-1.5, PN-0.3-2.0, and PN-0.3-2.5 exhibited fracture energies that were 137.24%, 166.75%, 185.49%, 179.75%, and 178.81% larger, respectively, than that of the C0 and PN-0.5-0.5, PN-0.5-1.0, PN-0.5-1.5, PN-0.5-2.0, and PN-0.5-2.5 exhibited fracture energies that were 420.04%, 451.31%, 417.81%, 317.11% and 292.75% larger, respectively.

From the test results, it can be seen that the NPFRCC specimen with 0.5% fiber admixture has a stronger restraining effect on the fracture deformation of concrete to prevent the expansion of internal cracks due to the presence of more fibers, which increases the energy consumption of the specimen at fracture during the damage process, and at the same time, the admixture of NS also enhances the adhesion in the region of the fracture path to strengthen the fracture energy. At the same fiber admixture, a moderate amount of NS (wt% > 1.5%) can promote hydration to enhance strength. At the same number of freeze–thaw cycle cycles, the 0.5% fiber specimen group had more fibers to provide residual strength after destabilizing damage occurred during the fracture process, so they had a larger area of the *P-COMD* curve and higher fracture energy. As the number of freeze–thaw cycles increased, the effect of concrete frost resistance on the fracture energy of the specimens gradually increased, and the PN-0.5-1.0 group was able to maintain higher fracture energy after several freeze–thaw cycles.

## 4. Conclusions

This study investigated the durability and mechanical properties of cementitious materials with different contents of NS and PVA fibers before, during, and after saline freeze–thaw cycling. The following conclusions were drawn from the results.

(1) The freeze–thaw cycle damage to the matrix occurred from the outside to the inside of each specimen, increased with the number of applied freeze–thaw cycles, and accelerated significantly after 50 freeze–thaw cycles. The PVA fibers provided a limited enhancement of freezing resistance but significantly improved the mechanical properties of the matrix through the bridging effect; the NS significantly improved the freezing resistance of the specimens by increasing the matrix density and the cohesive force on the fibers. Thus, the fibers and NS worked together to improve the freeze–thaw resistance and mechanical properties of NPFRCC specimens.

(2) The durability of NPFRCC specimens increased and then decreased with increasing NS and PVA fiber content, i.e., an optimal balance was found between the resistance to saline freeze–thaw cycles and the improvement of mechanical properties. Compared with the control group, the mass loss rate, dynamic modulus of elasticity, and compressive resistance of the PN-0.5-1.0 test group were improved by 71.98%, 62.23%, and 80.14% after 100 freeze–thaw cycles, respectively.

(3) The incorporation of PVA fibers significantly improved the fracture toughness of the NPFRCC specimens, but its enhancement of crack initiation toughness was limited; the crack initiation toughness, destabilization toughness, and fracture energy of PN-0.5-1.0 were enhanced by 120.69%, 160.02%, and 451.31%, respectively, compared to the control group after 100 freeze–thaw cycles considering the synergistic effect of NS. The incorporation of NS allowed the concrete to maintain high fracture toughness after freeze–thaw cycles, whereas PVA fiber incorporation had a significant effect on the improvement of concrete fracture toughness, and PVA fibers improved the fracture toughness of concrete more than NS.

## Figures and Tables

**Table 1:** Chemical composition of cement.

Composition (%)	CaO	SiO_2_	Al_2_O_3_	Fe_2_O_3_	MgO	SO_3_	R_2_O
Cement	62.31	21.05	5.50	3.92	1.72	2.66	0.47

**Table 2:** Physical properties of PVA fibers.

Specific Gravity	Diameter (μm)	Tensile Strength (MPa)	Length (mm)	Ductility (%)
1.3	40	1560	12	6.5

**Table 3:** Physical properties of NS.

Average Particle Size (nm)	Content (%)	Specific Surface Area (m^2^/g)	Bulk Density (g/cm^3^)	PH Value	Heating Reduction (%)	Burn Reduction (%)
30	99.5	180	0.055	6	1.0	1.0

**Table 4:** Mixing proportions of cementitious composites. (1 m^3^ concrete).

Group	Cement (kg/m^3^)	Quartz Sand (kg/m^3^)	Water (kg/m^3^)	Coarse Aggregate (kg/m^3^)	PVA Fiber (v%)	NS (wt%)	Water-Reducing Admixture (kg/m^3^)
C0 (control)	490	703	157	1100	0	0	8.3
P-0.1	490				0.1	0	
P-0.3	490				0.3	0	
P-0.5	490				0.5	0	
P-0.7	490				0.7	0	
P-0.9	490				0.9	0	
PN-0.3-0.5	487.55				0.3	0.5	
PN-0.3-1.0	485.10				0.3	1.0	
PN-0.3-1.5	482.65				0.3	1.5	
PN-0.3-2.0	480.20				0.3	2.0	
PN-0.3-2.5	477.75				0.3	2.5	
PN-0.5-0.5	487.55				0.5	0.5	
PN-0.5-1.0	485.10				0.5	1.0	
PN-0.5-1.5	482.65				0.5	1.5	
PN-0.5-2.0	480.20				0.5	2.0	
PN-0.5-2.5	477.75				0.5	2.5	

## Data Availability

The original contributions presented in the study are included in the article, further inquiries can be directed to the corresponding author.
